# Detecting long-lasting transients of earthquake activity on a fault system by monitoring apparent stress, ground motion and clustering

**DOI:** 10.1038/s41598-019-52756-8

**Published:** 2019-11-07

**Authors:** Matteo Picozzi, Dino Bindi, Aldo Zollo, Gaetano Festa, Daniele Spallarossa

**Affiliations:** 1 University of Naples Federico II, Naples, Italy; 2 Helmholtz Centre Potsdam, GFZ German Research Centre for Geosciences, Potsdam, Germany; 3 University of Genova, Genova, Italy

**Keywords:** Natural hazards, Seismology

## Abstract

Damaging earthquakes result from the evolution of stress in the brittle upper-crust, but the understanding of the mechanics of faulting cannot be achieved by only studying the large ones, which are rare. Considering a fault as a complex system, microearthquakes allow to set a benchmark in the system evolution. Here, we investigate the possibility to detect when a fault system starts deviating from a predefined benchmark behavior by monitoring the temporal and spatial variability of different micro-and-small magnitude earthquakes properties. We follow the temporal evolution of the apparent stress and of the event-specific residuals of ground shaking. Temporal and spatial clustering properties of microearthquakes are monitored as well. We focus on a fault system located in Southern Italy, where the M_w_ 6.9 Irpinia earthquake occurred in 1980. Following the temporal evolution of earthquakes parameters and their time-space distribution, we can identify two long-lasting phases in the seismicity patterns that are likely related to high pressure fluids in the shallow crust, which were otherwise impossible to decipher. Monitoring temporal and spatial variability of micro-to-small earthquakes source parameters at near fault observatories can have high potential as tool for providing us with new understanding of how the machine generating large earthquakes works.

## Introduction

How large is a fault strength? Do large earthquakes have a preparation phase preceding their occurrence? Finding answers to these key scientific questions is a challenge that the seismological community is called to address with the aim of mitigating seismic risk. The course seems clear: to understand the earthquake machine, and to single out precursors of stress concentrations, foreshocks and the nucleation of large earthquakes, the scientific community is currently following the methodological approach of creating multidisciplinary advanced research infrastructures, called Near Fault Observatories (NFOs). NFOs based on dense networks of multi-parametric sensors installed close to faults that continuously record high quality data related to the common underlying earth instability processes over a broad time interval (e.g., www.epos-ip.org); NFOs integrate cross-disciplinary information and are deemed to be promising means for monitoring the spatial and temporal evolution of faults mechanical properties, and possibly to unveil the earthquakes preparatory phase, faulting mechanisms, and the role of high pressure fluids in the crustal rupture processes^1–3^.

**Figure 1:**
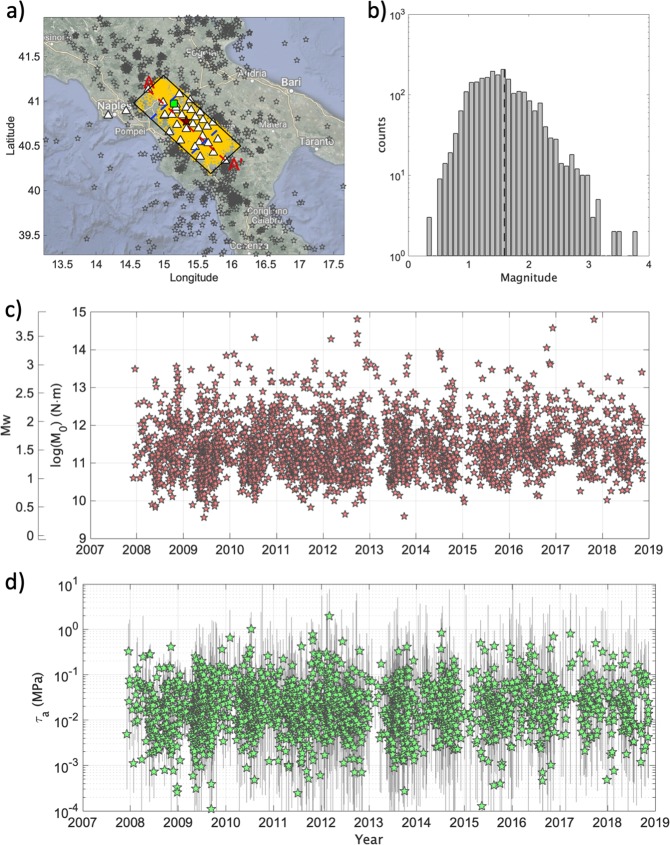
(**a**) Locations of the earthquakes considered in this study (yellow stars) and those recorded by ISNet but not considered (gray stars). ISNet seismic stations (white triangles). CO_2_ degassing site Mefite d’Ansanto (green square). Limits of the central sector (blue lines) and section A-A’ track (red line). (**b**) Distribution of Mw for the considered events, completeness magnitude Mc (dashed line). (**c**) M_0_ and Mw versus time. (**d**) Apparent stress ± 1 standard deviations shown as vertical bars. The map was made using MATLAB (R2016b; 9.1.0.441655; https://it.mathworks.com/, last accessed June 2019).

In this study, we consider one of the highest seismic risk areas in Europe, the Southern Apennines in Italy, a complex fault structure area with extensional kinematics characterized by a geodetic velocity of about 2 mm/year. This faults system generated several disastrous earthquakes in the last centuries, including the moment magnitude Mw 6.9, 1980 Irpinia earthquake^4,5^, which caused almost 3,000 fatalities. During the last ten years, southern Apennines have been monitored continuously and in real-time by the Irpinia Near Fault Observatory (INFO^6,7^), which includes ISNet (The Irpinia Seismic Network, http://isnet.fisica.unina.it) made up of 32 seismic stations covering an area of 100 × 70 km^2^, including the epicenter of the Mw 6.9, 1980 Irpinia earthquake. ISNet provides high quality recordings; the recorded magnitude ranges between Mw 0.3 and Mw 3.8 (Fig. 1a,b), being the completeness magnitude Mc equal to 1.6. ISNet’s characteristics fulfill the requirements^8^ for obtaining good quality micro-earthquake recordings, as shown by previous studies where source parameters estimates (e.g., M_0_, corner frequency *fc*, stress drop Δσ, radiation efficiency η_SW_) for hundreds of earthquakes^9^ were computed and a few microearthquake sequences^10^ identified.

The wealth of information carried by the nowadays available very large numbers of micro-to-small earthquakes is also pushing the seismological community to look for novel data analysis strategies with the aim to figure out the crustal strength spatial variation and its time evolution^11–14^. In this study, we analyze the temporal evolution of micro-to-small magnitude earthquakes at INFO, in southern Italy, in terms of apparent stress (τ_a_)^15^, between-event residuals (δBe)^16^ for the peak ground velocity (PGV) and applying a cluster analysis^17,18^.

τ_a_ was introduced in the 1960s^15^ and it is defined as the ratio between radiated energy (E_S_) and seismic moment (M_0_) multiplied by the rigidity of the source medium (μ)^19,20^ [i.e., τ_*a*_ = *μ E*_*S*_*/M*_0_]. Therefore, τ_a_ measures the amount of seismic energy radiated per unit fault area (*A*) and unit fault slip (*D*)^21^ [i.e., τ_*a*_ = *(E*_*S*_*/A)/D*]. When interpreted also as the difference between the average stress loading ($\bar{\tau }$) and the average stress that resist fault slip (${\bar{\tau }}_{R}$), τ_a_ represents the stress amount causing seismic energy radiation^22^. High τ_a_ values are associated with either high average stress level or lower frictional dynamic stress (i.e., conditions which may lead to material failure or facilitate seismic slip, respectively^23^); and, hence, τ_a_ can be used as a proxy for the crustal strength. Several recent studies used micro-to-moderate seismicity to compute τ_a_ for different purposes such as: relating τ_a_ to the stress causing earthquake fault slip^22^; for the time-dependent seismic hazard assessment and earthquake forecast in China^24^; for the identification of the migrating stress front in rock mass during mining activities^25,26^. Moreover, the variability in the source parameters also affects the event-specific variability of ground shaking. In Probabilistic Seismic Hazard Assessment, the ground shaking generated by a specific earthquake is predicted using Ground Motion Prediction Equations (GMPEs)^27^. Since most GMPEs model the source scaling considering only Mw, the ground shaking variability observed for earthquakes having the same Mw but showing differences in other source parameters (e.g., rupture velocity and stress drop, Δσ) is mapped to the between-event residual distribution δBe^16^ (i.e., which is the average discrepancy for a given event of the observations at different stations with respect to the median prediction). Recent studies^28–31^ highlighted the correlation between Δσ variability and δBe computed at high frequencies. Therefore, along with τ_a_, we also monitor the temporal variability of δBe.

Beside monitoring τ_a_ and δBe, we studied the observed seismicity applying a cluster analysis. Clustering analyses^17^ are considered essential elements for identifying the existence of different event populations (e.g., foreshocks, aftershocks, swarms, etc.), which can allow to better understand the seismic stress redistribution into the crust and the earthquake physics. The nearest-neighbor approach^17,18^ exploits the earthquake time-space-magnitude information to derive a generalized distance η between pairs of earthquakes, which can be used to isolate clusters from the background seismicity.

We considered about 2300 local earthquakes recorded by INFO over the last ten years. Inspired by previous works^32,33^, we mapped the heterogeneity distribution of τ_a_ over the Irpinia fault system. The source parameters M_0_ and E_S_ used to derive τ_a_ are obtained by applying the same approach used for the 2016–2017 Central Italy seismic^34,35^. Then, focusing on the more densely instrumented central sector of the Irpinia fault (i.e., a segment 60 km long), where the Mw 6.9, 1980 earthquake occurred, we studied the temporal evolution of τ_a_, δBe and η considering small and moderate magnitude earthquakes and looking for trends which are coherent among the parameters. To achieve this goal, we compared the cumulative of the considered parameters with the cumulative of a synthetic time series constructed assuming that the monitored parameters are constant for all events and equal to the median of their distributions. It is worth noting that, in order to define our monitoring strategy, we applied a retrospective approach; the prospective validation of the proposed methodology is ongoing considering the real-time data acquired at INFO. In the following, we refer to the synthetic time series as ‘reference model’, and they represent our benchmarks for the background fault activity. We therefore studied the difference between the cumulative experimental and synthetic series over time, which we define as residual apparent stress, Δτ_a_, and residual nearest-neighbor distance, Δη. Conversely, being δBe a parameter with a zero-average distribution, we simply considered its cumulative in time, ΣδBe. This approach allowed us to detect variations in all three studied parameters lasting few months and culminating with two Mw 3.5 earthquakes (i.e., occurred in the proximity of the Mw 6.9, 1980 earthquake’s hypocenter).

## Results

### τ_a_ spatial distribution over the Irpinia fault system

We examined 2336 earthquakes with M_w_ between 0.3 and 3.8 occurred within a buffer of +/− 30 km along the Irpinia fault system using hypocenter locations of the network bulletin from 2008 to 2018 (Fig. 1a–c). Uncertainties in the events locations are mostly within 1 km both horizontally and vertically (i.e., the median error in location is ~0.5 km, Fig. S1). We assessed E_S_ and M_0_ by extracting from the S-wave time window proxies for these two source parameters and correcting them for attenuation along the path^34,35^ (see “Method” section). So far, applications where E_S_ is estimated for small magnitude earthquakes has been rather limited, mainly due to difficulties in its estimation from band-limited recordings^36^. E_S_ can be computed either in the spectral or time domain^23,37^. Robust E_S_ estimates can be achieved only when recordings at short distances from dense networks of high quality instruments deployed with good azimuthal coverage around the source are available^8^.

**Figure 2:**
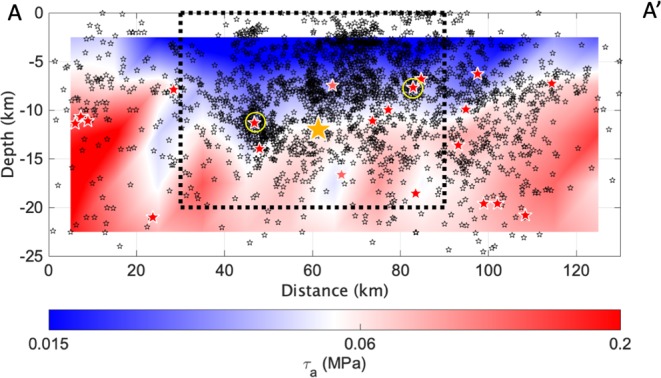
Contour of τ_a_ along the section A-A’ in Fig. (1a) derived considering Mw < 3.0 micro-earthquakes (black stars). Hypocenter of the Mw 6.9, 1980 Irpinia earthquake (yellow star) and of events with Mw > = 3.0 (stars colored per τ_a_). The two Mw 3.5 earthquakes discussed in the framework of the temporal analyses are indicated by yellow circles.

The linear scaling between the logarithm of E_S_ and M_0_ holds over 5 orders of magnitude for seismic moment (Fig. S2), and the scaled energy (i.e., E_S_/M_0_) variations with M_0_ well compares to previous results obtained for several tectonic areas^38^, and in particular for Central Italy^35^ (Fig. S3). We derived τ_a_ accounting for the depth variations of the crustal shear modulus μ, according to the 1D velocity model calibrated for the investigated area^39^. Figure (1d) shows as τ_a_ varies between 10^−3^ MPa and about 2 MPa over the decade of observations without, at a first glance, any clear trend. We analyzed the spatial distribution of τ_a_ along a section parallel to the Irpinia fault system dividing the fault into a 14 × 7 array of subfaults having a length of 10 km along the strike and a down-dip width of 5 km. Within each subfault, we stack the τ_a_ in logarithmic scale considering only events with Mw < 3.0, we compute the average τ_a_ value, and we assign it to the center of the subfault patch. Figure (2) shows that spatial interpolation of τ_a_ is characterized by large lateral and depth variations. τ_a_ value smaller than about 0.06 MPa (i.e. the average τ_a_) are confined to depths between 10 km and 15 km in the central fault sector (i.e., please note that the Mw 6,9, 1980 Irpinia earthquake nucleated at ~12 km), and to depths shallower than about 5 km in both the northern and southern fault sectors.

The distribution of hypocenters of events with Mw larger than 3.0 is also peculiar (i.e., please note that they are colored per their τ_a_ value in Fig. 2, and we remind they were not used to create the τ_a_ section). In the northern and southern portion of the fault, they are mostly located within areas with large τ_a_, whereas in the central sector, they are mostly distributed along the τ_a_ = 0.06 MPa boundary.

### τ_a_ temporal evolution

Several previous studies^40–42^ showed that the northern and southern Irpinia sectors are characterized by a complex tectonic, as resulting from the overlap of a strike-slip regime (i.e., related to the Benevento and Potenza faults to the north and to the south, respectively) with the extensional regime dominating the central sector of the Irpinia fault. Since such tectonic complexities would require a specific interpretation of the results for each sector, we restrict our interest to the more densely instrumented central sector of the Irpinia fault for studying the time evolution of τ_a_. This sector extends for 60 km, and it includes the area where the Mw 6.9, 1980 earthquake occurred. Furthermore, aiming to avoid possible biases due to variation in the number of stations during the last decade, we considered only earthquakes above the completeness magnitude at INFO (i.e., Mc = 1.6), thus in total, 928 earthquakes are considered.

**Figure 3:**
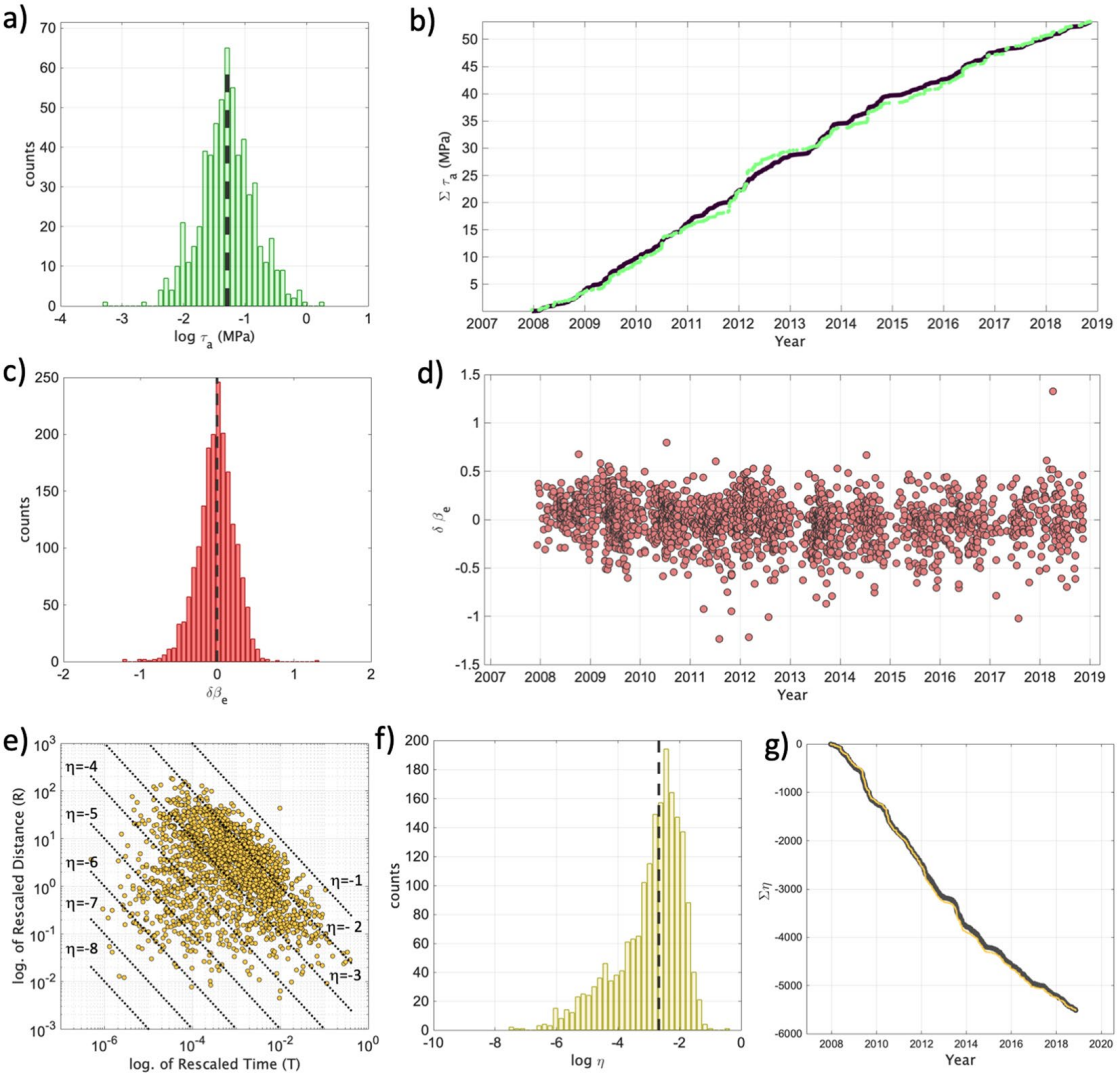
(**a**) Distribution of τ_a_ and median value (${\tilde{\tau }}_{a}$, dashed black line). (**b**) Cumulative of τ_a_ (green) and of ${\tilde{\tau }}_{a}$ (black). (**c**) Distribution of the between-event residuals (δBe) computed for PGV and median value (dashed black line). (**d**) δBe versus time. (**e**) 2D distribution of the rescaled time T and distance R. The dotted lines correspond to different values of the nearest-neighbor distance η. (**f**) Distribution of η and median value $\tilde{\eta }$ (dashed black line). (**g**) Cumulative of η (yellow) and of $\tilde{\eta }$ (black).

**Figure 4:**
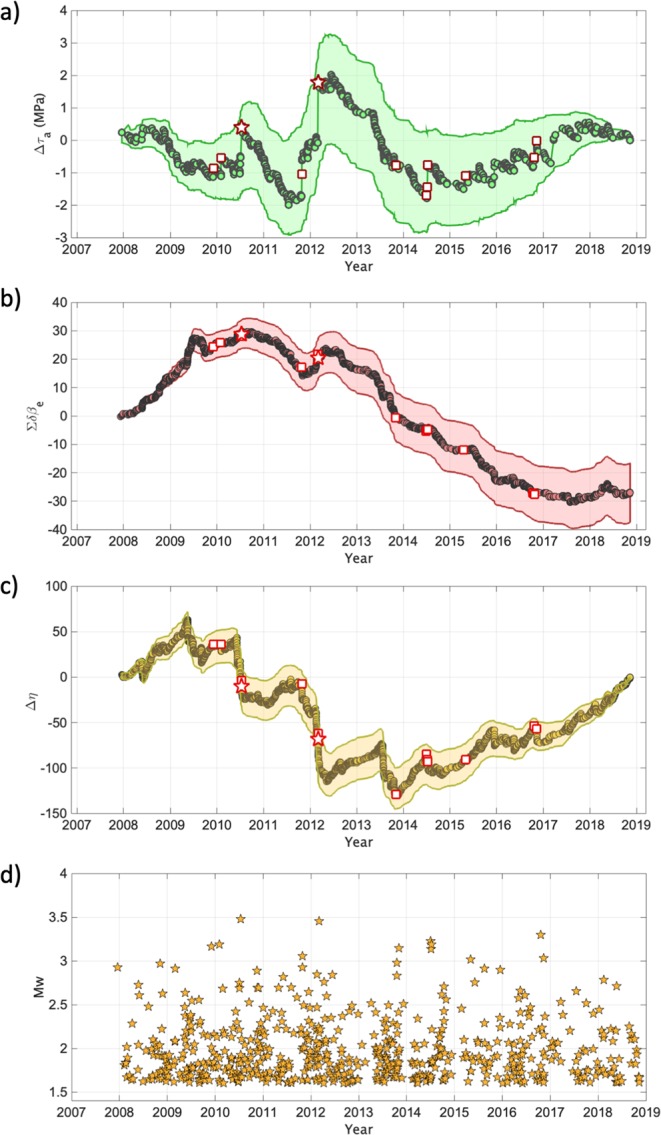
(**a**) Temporal variability of the residual apparent stress (Δτ_a_). Events with magnitude below Mw 3.0 (circles), 3.0 ≤ Mw < 3.5 (white square with red contour), and those with Mw = 3.5 (white stars with red contour). The light green area indicates the median +/− one standard deviation range as resulting from bootstrap analysis. (**b**) The same as (**a**), but for ΣδBe. (**c**) The same as (**a**), but for Δηs. (**d**) Distribution of Mw for the considered events versus time.

Figure (3a,b) shows the histogram of the τ_a_ values, their cumulative distribution (i.e., Στ_a-obs_) and the cumulative distribution of the reference model (i.e., Στ_a-syn_, the model for constant apparent stress). We investigated the deviations of Στ_a-obs_ from Στ_a-syn_ (Fig. 3b) by computing their residuals Δτ_a_ = Στ_a-obs_ - Στ_a-syn_. Events are represented in the Δτ_a_ curve (Fig. 4a) with different symbols: green dots for Mw < 3.0; white squares for Mw is the range between 3.0 and 3.4; white stars for Mw > 3.4. Considering the average magnitude uncertainty for the earthquakes in the three classes (i.e., ΔMw = 0.43 for Mw < 3.0; ΔMw = 0.25 for 3.0 <  = Mw < 3.4; ΔMw = 0.24 for Mw >  = 3.4), we cannot rule out that some events could have been misclassified. For the sake of simplicity, hereinafter earthquakes with magnitude equal to Mw 3.5 are also referred to as mainshocks. Finally, we evaluated the uncertainty associated to Δτ_a_ through bootstrap analysis^43^ using 2000 realizations. This means that, for each realization, we randomly resample the τ_a_ population, we compute the median value and the two cumulative curves from which Δτ_a_ is derived. Finally, the 2000 Δτ_a_ curves are used to estimate the uncertainty. Figure (4a) shows that the uncertainty does not mask the time evolution of the studied parameter. Looking at the trend of Δτ_a_ and at the occurrence of the largest magnitude events (Fig. 4a), it is striking the presence of some peculiar trends. During the period 2008–2010, Δτ_a_ steadily decreased, accumulating a reduction of about 1 MPa. Starting from the begin of 2010 two similar Δτ_a_ cycles developed (i.e., the first from 2010 to 2012, the second from 2012 to 2014), characterized by: (i) a few months lasting increase in Δτ_a_ (i.e., accumulating about ~1.5 MPa and about ~4 MPa, respectively), (ii) the occurrence of Mw 3.5 at the end of each Δτ_a_ increasing period, iii) a slow decrease in Δτ_a_ lasting for about two years. The increase of Δτ_a_ before the two Mw 3.5 lasted about 8 and half months before the first event, and about 4 and half months before the second one. The decreasing phases lasted one year and a half and two years and half, respectively. After these two cycles, since 2014 we observe a rather slow and regular increase in Δτ_a_, during which seven earthquakes with magnitude between Mw 3.0 and 3.4 have occurred.

### δBe temporal evolution

The strategy applied to detect temporal patterns in the source properties, which could hint towards the identification of key dynamic characteristics of rupture processes, was to monitor deviations from a reference model characterized by apparent stress constant for all events. Since the variability affecting source parameters leaves an imprint in the variability of the ground shaking generated at the same distance by earthquakes sharing the same magnitude, we apply also a strategy based on monitoring the ground motion residuals computed with respect to a reference ground motion prediction equation. Also for the ground motion residuals we apply a retrospective approach: we calibrate the reference GMPE over the last 10 years of data (see Method) and then we investigate the temporal distribution of the so called inter-event residuals δBe^16^. Since the source scaling of the GMPE is controlled mainly by magnitude, differences in source parameters other than the magnitude are mapped to the residuals. Previous works^28–31,44^ showed that δBe values computed for the peak ground acceleration (PGA) or velocity (PGV) are correlated with stress drop variability. In this study, the GMPE is calibrated for the logarithm of PGV. The inter-event residuals δBe describe a normal distribution centered around zero (i.e., on average the GMPE predicts the observed PGV values without any bias) and no trends with magnitude are observed (Fig. S5). Figure (3a,b) show the distribution of δBe and its temporal evolution, respectively. As for τ_a_, no clear temporal trends in δBe are observed. Therefore, we studied the temporal evolution of its cumulative, ΣδBe (Fig. 4b), interpreted in comparison with Δτ_a_. The uncertainty associated to ΣδBe is computed by bootstrapping the dataset^43^ considering 2000 replications, as for Δτ_a_. Interestingly, locally ΣδBe shows a (positive) trend over the two periods culminating with the two Mw 3.5 earthquakes, where also Δτ_a_ increase, strengthening the hypothesis that a background process influences the dynamical characteristics of microseismicity rupture process in the investigated fault system. Or, in other words, anomalies in the temporal distribution of τ_a_ leave an imprint also in the ground shaking variability.

### η temporal evolution

We analyzed the statistical features of the recorded earthquakes to verify if, beyond the background seismicity, clustered subpopulations of events could be identified. To this aim, we applied the nearest-neighbor approach^17,18^ and computed the generalized distance between pairs of earthquakes, η (see Method).

Figure (3e) shows the rescaled time and space components (T, R) of η, whereas each point in the plot represents an event. The cloud of points is distributed mainly between η = −1 and −3. The distribution of η is unimodal (with median value $\tilde{\eta }$ equal to −2.7; Fig. 3f), and we cannot identify any clear cluster from the background seismicity^18^. Following the analyses done for Δτ_a_ and ΣδBe, which suggested the presence of cyclic variations in the earthquakes source properties, we investigated the temporal evolution of η. Figure (3g) shows the cumulative of η compared to the reference cumulative constructed assuming η to be constant and equal to its median value, $\tilde{\eta }$, whereas Fig. (4c) shows the difference of the two cumulative functions $\Delta {\rm{\eta }}=\Sigma {\rm{\eta }}-\Sigma \tilde{\eta }$ versus time. The uncertainty associated to Δη is again derived by a bootstrap analysis^43^ using 2000 realizations.

It is worth noting the presence of significant (negative) trends in Δη before the occurrence of the two Mw 3.5 (Fig. 4c), which seems well related in time with variations of Δτ_a_ and ΣδBe. However, while Δτ_a_ is related to dynamic properties of the earthquakes, for Δη we still miss a physical interpretation. By construction, the cumulative series Ση and Σ$\tilde{\eta }$ have the same intercurrence time *t* (See Eqs 5–7 in section ‘Method’). Furthermore, changes in the earthquake distance *r* (such us to justify a decrease in Δη) should appear as multi-modal distribution of η (i.e., a mode for each cluster); while, we see for η an unimodal distribution. Hence, both *t* and *r* can in first approximation be neglected. Looking at Eqs (5) and (6), two parameters that might play a role on the variations of Δη are the fractal dimension *d* and the parameter of the Gutenberg-Richter parameter *b*. Since a correlation between *d* and the *b* is reported in literature e.g.^45,46^, we focused only on the relation between *d* and Δη. To verify if a relation between changes in *d* and Δη does exist, we used the approach proposed by Grassberger and Procaccia (1983)^47^ to estimate *d*. Following a previous study^45^, we used sliding windows of 200 events, shifted by one event per time. Figure (S5) shows the evolution of the fractal dimension of hypocenters with time. Despite, in our opinion, the analysis for estimating *d* is less robust than the procedure for computing η, mainly because the former implies a best-fit procedure, we consider remarkable the good correlation between results from the two analyses (i.e., *d* and Δη). Indeed, also *d* shows a decrease starting from the 2009, before the first Mw 3.5 earthquakes, and a second one during the 2011, before the second Mw 3.5 (Fig. S5).

To highlight the association between changes in Δτ_a_, Δη and δBe, we performed a correlation analysis. Figure (S6a) shows the comparison of the normalized Δτ_a_ and Δη time series together with splines used to guarantee a homogeneous sampling and to emphasize low frequency trend. After the linear trend removal (Fig. S6b), the splines are used to compute the Spearman’s correlation coefficient on moving windows of 50 points and 90% of overlap. The distribution of Spearman’s correlation coefficients shown in Fig. S6c, has a maximum at −1, indicating that Δτ_a_ and Δη are strongly anti-correlated. A similar analysis was carried out also for Δτ_a_ and δBe (Fig. S7). In this case, the Spearman’s correlation coefficients indicate a strong positive correlation (Fig. S7c).

## Discussion

The seismic velocity and attenuation structure in the Irpinia region is well-known^48,49^. This region is affected by crustal extension^42^, generating a seismicity characterized by small to moderate magnitude events marked by strong earthquakes, as the Mw 6.9, 1980 Irpinia earthquake. The seismic active rock volume consists of the Apulian Platform carbonates and its basement. The 3-D images of the Irpinia fault system in P and S waves velocity^48^ indicate a highly fractured rock volume rich of fluid in the uppermost 15 km, with background seismicity driven by pore pressure changes in fluid-filled cracks. Further important insights on the underground structure were provided by the 3-D attenuation images^49^, showing Q_P_ anomalies well correlated to the Mw 6.9, 1980 Irpinia earthquake nucleation area. Moreover, Q_S_ images highlight a strong lateral variation associated with the contrast between the thick, slower and high attenuation Miocene basin sediments and the Apulian Platform carbonates^50^.

The spatial distribution of τ_a_ well complement the velocity and attenuation images. Low τ_a_ values in the shallower portion (i.e. within ~15 km) of the central sector in the Irpinia fault system correspond to the highly fractured carbonate rocks. The increase of τ_a_ at about 15 km, a depth compatible with the nucleation area of the Mw 6.9, 1980 Irpinia earthquake, likely marks the contrast between carbonates with the underlying basement. In both the northern and southern portions of the analyzed fault system, high τ_a_ values are observed at shallower depths (i.e., ~5 km), confirming the high heterogeneity and complexity of the Irpinia region. In these external sectors, indeed, focal mechanisms^40–42^ indicate the presence of faults with different kinematic and suggest the intersection between the Irpinia fault system and the Benevento strike-slip zone at north and the Potenza strike-slip zone at south. In support to this hypothesis, the 2012 Benevento and the 1990–1991 Potenza seismic sequences (i.e., respectively, in the northern and southern sectors of the investigated area) present strong similarities: have similar kinematics, are both crustal but have occurred at relatively high depth (~between 15 and 25 km) well below the Apulian carbonate units that represents the upper crust level where the Mw 6.9, 1980 earthquake’s hypocenter is located^40,41^.

The temporal evolution of Δτ_a_ suggested the presence of cyclic variations in the earthquakes source properties and stimulated our curiosity on its physical interpretation. As discussed in previous studies, τ_a_ can be defined as the product between the seismic radiation efficiency, ρ, and the shear stress causing fault slip, τ_S_ (i.e., τ_*a*_ = *ρ* · τ_*S*_)^22,51^. In the Irpinia fault region ρ is low and, in general, corresponds to a small fraction of the energy is spent for creating new fractures^9^. Indeed, the Savage and Wood efficiency^52^ ρ_sw_ for Irpinia microearthquakes (i.e., please note that ρ_sw_ is one-half of ρ and it represents the link between apparent stress and static stress drop, Δσ, commonly used by seismologists, τ_*a*_ = *ρ*_SW_ · Δσ) remains almost constant over 4 orders of magnitude^9^ (i.e., with median ρ_sw_ = 0.06). Therefore, we consider reasonable to assume ρ being constant. Hence, Δτ_a_ can be approximated as follows:

1 $$\Delta {\tau }_{a}={\sum }_{i}^{n}({\tau }_{ai}-\tilde{{\tau }_{a}})={\sum }_{i}^{n}({\rho }_{i}{\tau }_{s}-\tilde{\rho }\tilde{{\tau }_{s}})\approx \Delta {\tau }_{s}$$

where the cumulative for the radiation efficiency is approximated with the cumulative of the average radiation efficiency $\sum {\rho }_{I}=\sum \tilde{\rho }.$ Under this condition, Δτ_a_ can be considered a proxy for Δτ_S_, the fault strength loss. Studying the temporal evolution of Δτ_a_ is akin to study the temporal evolution of the Gutenberg-Richter law b parameter^53^. Indeed, while single events provide information of the stress acting in specific location of the fault plane (i.e., for instance by their stress drop), the b parameter captures their collective behavior and is considered well representative of the differential stress acting on the whole fault^54^. Similarly, τ_a_ for single events represents the stress causing seismic energy radiation, but performing the temporal analysis over Δτ_a_, we highlight the collective behavior of the earthquakes, and we provide a first order estimate of the crustal strength for the central sector of the Irpinia fault system. From our analyses, Δτ_a_ increases between about ~1.5 MPa and ~4 MPa observed in the periods preceding the Mw 3.5. Previous studies analyzing microearthquakes in the Irpinia area estimated a median stress drop varying from 1.4 MPa^9^ to 3.9 MPa^10^, well in agreement with Δσ = 3.5 MPa estimated for the Mw 6.9, 1980 Irpinia earthquake^55^.

Since the inter-event residuals δBe absorb event-specific component of the ground motion variability not controlled by the seismic moment, the consistency between the trends observed for these two parameters during periods lasting a few months before the two Mw 3.5 earthquakes lead us to hypothesize the existence of background processes influencing the dynamical characteristics of microseismicity rupture process. One further, independent, piece of the puzzle is the decrease of Δη (Fig. 4c), which we have shown likely be indicative of a decrease in the fractal dimension *d* (Fig. S4b). Empirically, values of *d* close to 2, as for the first Mw 3.5 earthquake, or slightly below it (i.e., ~1.5 as for the second Mw 3.5), suggest that the hypocentres of the earthquakes tend to be progressively distributed over a two-dimensional fault plane before the mainshocks^56^. Therefore, we can interpret the increase of Δτ_a_ and ΣδBe, combined with the reduction of Δη, as the result of a progressive activation of seismic asperities in the fault plane that have higher Δσ than the surrounding area. The time with which this process take place (i.e., few months) suggest the presence of a slow aseismic force. On the nature of this force, we can provide only a guess. Upscaling procedures based on velocity and attenuation^49^ lead to estimate a range of porosity in carbonates (i.e., 4 to 5%) and the fluid composition consisting in brine-CO_2_ and/or CH_4_-CO_2_. The CO_2_ earth degassing in central and southern Italy is largely documented^57,58^. In particular, the Mefite d’Ansanto located in the Irpinia region (Fig. 1a) is considered one of the largest natural emission from non-volcanic environment of low-temperature CO_2_ rich gases ever measured^58^. The spatial distribution of the CO_2_ degassing areas and seismicity in the Apennines provides an interpretative scheme for the seismicity generation^57^. The large upwelling of mantle fluids in the Tyrrhenian hinterland infiltrate upward through interconnected fractures generated by extensional tectonic regime and generate high CO_2_ domains in the shallower crust. Whereas fractures are well interconnected, degassing at the surface is observed. On the contrary, in the Apennines, the arrangements of deeper thrust and low-angle normal faults create verging structure where CO_2_ may accumulate and generate overpressurized reservoirs at depths ranging from few kilometres to about 15 km^57^. In the case of the 2009 L’Aquila earthquake in Italy (Mw 6.3, central Apennines), strong evidences for the presence of overpressured fluids near the foreshocks and mainshock were provided^11,59,60^, suggesting that they contributed to the mainshock rupture. Similar conclusions were proposed also for two earthquakes of magnitudes 5.7 and 6 occurred in 1997 in the northern Apennines^61^. In this framework, we can interpret our results as related to CO_2_-brine accumulated and sealed within the rock volume. When the pressure in the deep CO_2_-brine reservoir increases, the seismicity tends to progressively be distributed over two-dimensional fault planes; the process culminates with Mw ≥ 3.5 earthquakes, which evidently allows for the creation of way outs large enough for the fluids migration towards the surface. The sequences are then followed by a slow decrease in the stress level (i.e., Δτ_a_ decreases) until a new stress loading cycle starts.

In future, it will be certainly important to focus on areas where large magnitude events have occurred, to investigate if a relation between the rate and amplitude of the Δτ_a_ anomalies and the earthquake magnitude does exist. In conclusion, in our vision for NFOs, we think that studying the microseismicity and small-to-moderate earthquakes through a wide set of parameters (e.g., including also focal mechanisms^11^ and medium changes^59^) can have high potential as tool for monitoring the evolution of seismic sequences, identifying the preparatory phase of large earthquakes and providing us with new understanding of how does work the machine generating earthquakes.

## Methods

### Data processing

We adopted a procedure proposed for the Central Italy seismic sequence^34,35^, where M_0_ and E_S_ are derived from two proxies measured on direct S-waves: the peak displacement (PD_S_) and the cumulative squared velocity (IV2_S_), respectively. Processing was carried out as follows: P-waves have been hand-picked on three-component ground velocity recordings sampled at 125 Hz, while S-waves arrival has been estimated considering location and a 1D velocity model optimized for the Irpinia area; Butterworth band pass pre-deconvolution filter with high and low pass corner frequencies at 0.5 Hz and 40 Hz, respectively; Instrumental correction and computation of acceleration, velocity and displacement. The parameters PD_S_ and IV2_S_ are computed considering a time window starting 0.1 s before the S-wave onset and ending at different percentages of the total energy as a function of the source to site distance R: (i) 90 per cent when R < 25 km; (ii) 80 per cent when 25 km < R < 50 km; (iii) 70 per cent when R > 50 km. For both PD_S_ and IV2_S_ calculations, we imposed a minimum time window length of 5 s and a maximum time window length of 20 s. For each recording, a signal-to-noise ratio was evaluated considering a pre-event noise window of the same length as the signal window. Finally, the values of the three components of ground motion are averaged for both PD_S_ and IV2_S_. Furthermore, a subset of data was extracted with the following criteria: hypocentral distance smaller than 60 km; events recorded by a minimum of 3 stations; the sum of SNR for the three components ≥ 200.

### Seismic moment and radiated energy estimation

From a database of 2336 earthquakes, source parameters (i.e., M_0_, *fc*, Δσ and η_SW_) were available^9^ for 216 events with magnitude ranging between M_w_ 1.2 and M_w_ 3.2. The dataset of 216 earthquakes was thus used to link the experimental IV2_S_ and PD_S_ to well-constrained M_0_ values and theoretical E_S_ (i.e., obtained from source parameters and the spectral integration^37^ of the squared theoretical Brune’s velocity spectrum for S-waves^60^) and to calibrate empirical attenuation models. Having assumed the Brune’s seismic source model, our E_S_ and M_0_ estimates are model dependent. We assumed a linear model, where the attenuation of average values of the proxy (i.e., either IV2_S_ or PD_S_) as a function of distance is expressed without assuming any a-priori functional form:

2 $$log[IV{2}_{S}({R}_{H})]=A+Blog({E}_{S})+{w}_{j}{C}_{j}+(1-{w}_{j}){C}_{j+1}$$

and

3 $$log[P{D}_{S}({R}_{H})]=D+Flog({M}_{0})+{w}_{j}{G}_{j}+(1-{w}_{j}){G}_{j+1}$$

where, the hypocentral distance R_H_ ranging between 5 km and 60 km is discretized into 12 bins (N_bin_) with equal width (i.e., 5 km); the index *j* = 1,..,N_bin_ indicates the *j-th* node selected such that R_H_ is between the distances r_j_ ≤ R_H_ < r_j+1_; the attenuation function is linearized between nodes r_j_ and r_j+1_ using the weights *w*, computed as *w*_j_ = (r_j+1_ − R_H_)/(r_j+1_ − r_j_). The minimum distance is fixed to 5 km given the lack of recordings at shorter distances. The coefficients of Eqs (2) and (3) are determined by solving the over-determined linear system in a least-square sense. The trade-off between A and C_j_, and between D and G_j_ is constrained by the condition to be zero at 10 km. It is worth noting that changing the node constrained to zero corresponds to changes of both the A and C_j_, as well as for D and G_j_, which compensate each other, so that the coefficient B and F in Eqs (2) and (3) are unaltered. The attenuation models obtained for the Irpinia area agree with those obtained for the Central Italy seismic sequence^35^ (Fig. S5). The attenuation models are reported in Table S1. The event E_S_ and M_0_ estimates per event, as well as the derived τ_a_ ones, are obtained by averaging over the recording stations.

### Between event residuals, δBe, estimation

We performed a linear regression analysis to model the PGV scaling with distance and magnitude by searching the best-fit parameters of the following equation:

4 $$\log ({\rm{PGV}})={\rm{A}}+{\rm{B}}1\cdot ({{\rm{M}}}_{{\rm{w}}}-{{\rm{M}}}_{{\rm{ref}}})+{\rm{B}}2\cdot {({{\rm{M}}}_{{\rm{w}}}-{{\rm{M}}}_{{\rm{ref}}})}^{2}+{\rm{C}}\cdot \,\log ({\rm{R}}),$$

where R is the hypocentral distance in km and PGV is in cm/s, and the reference magnitude M_ref_ is set equal to 1. Like previous studies^28,61–63^, we refer to a simple ground motion model, since we do not have the ambition to set up a GMPE for hazard calculation. Our aim is to monitor the variations with time of the between-event variability of ground motion. The result of the regression analysis is provided in Table (S2). The between-event residuals (δBe) are computed as the average difference, for any given event, between the PGV measured at different stations and the corresponding values predicted by the GMPE.

### Nearest-neighbor distance, η, analysis

The nearest-neighbor approach^17,18^ is based on the computation of the generalized distance between pairs of earthquakes, η, from the analysis of the time-space distances between pairs of earthquakes. The parameter η is derived computing the distances in time (i.e., Rescaled Time) and space (i.e., Rescaled Distance) between an event *i* and its parent *j* normalized by the magnitude of the parent event:

5 $${T}_{ij}={t}_{ij}{10}^{-b{m}_{i}/2}$$

6 $${R}_{ij}={({r}_{ij})}^{d}{10}^{-b{m}_{i}/2}$$

where, *m* is the magnitude (M_w_), *b* is the parameter of the Gutenberg-Richter law, *t* is the earthquake intercurrence time, *r* is the earthquake distance, and *d* is the fractal dimension. We fixed *b* = 1 and *d* = 1.6, following Zaliapin and Ben-Zion^64^, and we verified that, as claimed into that study, the cluster analysis is not much sensitive to the selection of precise parameter values.

Finally, η is defined as:

7 $$\log \,{{\rm{\eta }}}_{{\rm{ij}}}=\,\log \,{{\rm{R}}}_{{\rm{ij}}}+\,\log \,{{\rm{T}}}_{{\rm{ij}}}$$

(see Zaliapin and Ben-Zion, 2016^18^ for further details).

## Supplementary information

Supplementary Information

## References

[1] Malin PE (2018). Microearthquakes preceding a M4.2 Earthquake Offshore Istanbul. Scientific Reports.

[2] Sugan M, Kato A, Miyake H, Nakagawa S, Vuan A (2014). The preparatory phase of the 2009 Mw 6.3 L’Aquila earthquake by improving the detection capability of low-magnitude foreshocks. Geophys. Res. Lett..

[3] Allmann BP, Shearer PM (2007). Spatial and temporal stress drop variations in small earthquakes near Parkfield, California. J. Geophys. Res..

[4] Bernard P, Zollo A (1989). The Irpinia (Italy) 1980 earthquake: Detailed analysis of a complex normal fault. J. Geophys. Res..

[5] Cocco M (1999). The April 1996 Irpinia seismic sequence: evidence for fault interaction. J. Seismol..

[6] European Research Infrastructure On Solid Earth, https://www.epos-ip.org/data-services/community-services-tcs/near-fault-observatories.

[7] Vassallo M, Festa G, Bobbio A (2012). Seismic ambient noise analysis in southern Italy. Bull. Seismol. Soc. Am..

[8] Kwiatek G, Ben-Zion Y (2016). Theoretical limits on detection and analysis of small earthquakes. J. Geophys. Res..

[9] Zollo A, Orefice A, Convertito V (2014). Source parameter scaling and radiation efficiency of microearth- quakes along the Irpinia fault zone in southern Apennines, Italy. J. Geophys. Res. Solid Earth.

[10] Stabile TA, Satriano C, Orefice A, Festa G, Zollo A (2012). Anatomy of a microearthquake sequence on an active normal fault. Scientific Reports.

[11] Terakawa T, Zoporowski A, Galvan B, Miller SA (2010). High-pressure fluid at hypocentral depths in the L’Aquila region inferred from earthquake focal mechanisms. Geology.

[12] Shebalin P, Narteau C (2017). Depth dependent stress revealed by aftershocks. Nature Communications.

[13] Yamada T, Saito Y, Tanioka Y, Kawahara J (2017). Spatial pattern in stress drops of moderate- sized earthquakes on the Pacific Plate off the south-east of Hokkaido, Japan: implications for the heterogeneity of frictional properties. Progress in Earth and Planetary. Science.

[14] Yoshida K, Hasegawa A, Yoshida T (2016). Temporal variation of frictional strength in an earthquake swarm in NE Japan caused by fluid migration. J. Geophys. Res. Solid Earth.

[15] Wyss M, Brune JN (1968). Seismic moment, stress, and source dimensions for earthquakes in the California-Nevada region. J. Geophys. Res..

[16] Al Atik L (2010). The variability of ground-motion prediction models and its components. Seismol. Res. Lett..

[17] Zaliapin I, Gabrielov A, Keilis-Borok V, Wong H (2008). Clustering analysis of seismicity and aftershock identification. Phys. Rev. Lett..

[18] Zaliapin I, Ben-Zion Y (2016). Discriminating Characteristics of Tectonic and Human-Induced Seismicity. Bulletin of the Seismological Society of America.

[19] Choy GL, Boatwright J (1995). Global patterns of radiated seismic energy and apparent stress. J. Geophys. Res..

[20] McGarr A (1999). On relating apparent stress to the stress causing earth-quake fault slip. J. Geophys. Res..

[21] Mori J, Abercrombie RE, Kanamori H (2003). Stress drops and radiated energies of aftershocks of the 1994 Northridge, California, earthquake. J. Geophys. Res..

[22] McGarr A (1999). On relating apparent stress to the stress causing earth- quake fault slip. J. Geophys. Res..

[23] Kanamori H, Hauksson E, Hutton LK, Jones LM (1993). Determination of earthquake energy release and ML using TERRAscope. Bull. Seismol. Soc. Am..

[24] Wu Z, Jiang C, Zhang S (2017). Can apparent stress be used to time-dependent seismic hazard assessment or earthquake forecast? An ongoing approach in China. Pure Appl. Geophys..

[25] Brown, L. G. & Hudyma, M. R. Identifying a migrating stress front using apparent stress for an unplanned rock mass cave. *Proceedings of the Fourth International Symposium on Block and Sublevel Caving, Australian Centre for Geomechanics*, Perth, 565–576 (2018).

[26] Carusone, O. & Hudyma, M. Variations in apparent stress and energy index as indicators of stress and yielding around excavations. *Proceedings of the First International Conference on Underground Mining Technology, Australian Centre for Geomechanics*, Perth, 205–218 (2017).

[27] Douglas J, Edwards B (2016). Recent and future developments in earthquake ground motion estimation. Earth-Science Reviews.

[28] Oth A, Miyake H, Bindi D (2017). On the relation of earthquake stress drop and ground motion variability. J. Geophys. Res. Solid Earth.

[29] Ameri G, Drouet S, Traversa P, Bindi D, Cotton F (2017). Toward an empirical ground motion prediction equation for France: Accounting for regional differences in the source stress parameter. Bull. Earthquake Eng..

[30] Bindi D, Cotton F, Spallarossa D, Picozzi M, Rivalta E (2018). Temporal Variability of Ground Shaking and Stress Drop in Central Italy: A Hint for Fault Healing?. Bulletin of the Seismological Society of America.

[31] Bindi D, Picozzi M, Spallarossa D, Cotton F, Kotha SR (2019). Impact of Magnitude Selection on Aleatory Variability Associated with Ground-Motion Prediction Equations: Part II-Analysis of the Between-Event Distribution in Central Italy. Bulletin of the Seismological Society of America.

[32] McGarr A, Fletcher JB (2000). A method for mapping apparent stress and energy radiation applied to the 1994 Northridge earthquake fault zone. Geophysical Research Letters.

[33] McGarr A, Fletcher JB (2002). Mapping apparent stress and energy radiation over fault zones of major earthquakes. Bull. Seism. Soc. Am..

[34] Picozzi M (2017). Rapid determination of P wave- based energy magnitude: Insights on source parameter scaling of the 2016 Central Italy earthquake sequence. Geophys. Res. Lett..

[35] Picozzi M, Bindi D, Spallarossa D, Di Giacomo D, Zollo A (2018). A rapid response magnitude scale for timely assessment of the high frequency seismic radiation. Scientific Reports.

[36] Ide S, Beroza GC (2001). Does apparent stress vary with earthquake size?. Geophys. Res. Lett..

[37] Izutani Y, Kanamori H (2001). Scale-dependence of seismic energy-to-moment ratio for strike- slip earthuakes in Japan. Geophys. Res. Lett..

[38] Kanamori H, Brodsky EE (2004). The physics of earthquakes. Rep. Prog. Phys..

[39] Matrullo E, De Matteis R, Satriano C, Amoroso O, Zollo A (2013). An improved 1-D seismic velocity model for seismological studies in Campania-Lucania region (Southern Italy). Geophys. J. Int..

[40] Adinolfi Guido Maria, De Matteis Raffaella, Orefice Antonella, Festa Gaetano, Zollo Aldo, de Nardis Rita, Lavecchia Giusy (2015). The September 27, 2012, ML 4.1, Benevento earthquake: A case of strike-slip faulting in Southern Apennines (Italy). Tectonophysics.

[41] Boncio P, Mancini T, Lavecchia G, Selvaggi G (2007). Seismotectonics of strike-slip earthquakes within the deep crust of southern Italy: Geometry, kinematics, stress field and crustal rheology of the Potenza 1990-1991 seismic sequences (Mmax 5.7). Tectonophysics.

[42] De Matteis R, Matrullo E, Stabile TA, Rivera LA, Zollo A (2012). Fault delineation and regional stress direction from the analysis of background microseismicity in southern Apennines, Italy. Bull. Seismol. Soc. Am..

[43] Efron B (1979). Bootstrap methods: another look at the jackknife. Ann. Stat..

[44] Baltay AS, Hanks TC, Abrahamson NA (2017). Uncertainty, variability, and earthquake physics in ground-motion prediction equations. Bulletin of the Seismological Society of America.

[45] Henderson JR, Barton DJ, Foulger GR (1999). Fractal clustering of induced seismicity in The Geysers geothermal area, California. Geophys. J. Int..

[46] Oncel AO, Alptekin O, Main IG (1995). Temporal variations of the fractal properties in the western part of the North Anatolian fault zone: possible artifacts due to improvements in station coverage. Nonlin. Proc. Geophys..

[47] Grassberger P, Procaccia I (1983). Measuring the strangeness of strange attractors. Physica.

[48] Amoroso O, Ascione A, Mazzoli S, Virieux J, Zollo A (2014). Seismic imaging of a fluid storage in the actively extending Apennine mountain belt, southern Italy. Geophys. Res. Lett..

[49] Amoroso O (2017). From velocity and attenuation tomography to rock physical modeling: Inferences on fluid-driven earthquake processes at the Irpinia fault system in southern Italy. Geophys. Res. Lett..

[50] Ascione A, Mazzoli S, Petrosino P, Valente E (2013). A decoupled kinematic model for active normal faults: Insights from the 1980, MS = 6.9 Irpinia earthquake, southern Italy. Geol. Soc. Am. Bull..

[51] Beeler NM, Wong TF, Hickman SH (2003). On the expected relationships among apparent stress, static stress drop, effective shear fracture energy, and efficiency. Bull. Seismol. Soc. Am..

[52] Savage JC, Wood MD (1971). The relation between apparent stress and stress drop. Bull. Seismol. Soc. Am..

[53] Gutenberg B, Richter CF (1942). Earthquake magnitude, intensity, energy, and acceleration. Bull. Seismol. Soc. Am..

[54] Scholz CH (2015). On the stress dependence of the earthquake b value. Geophysical Research Letters.

[55] Deschamps A, King GCP (1983). The Campania-Lucania (southern Italy) earthquake of 23 November 1980. Earth Planet. Sci. Lett..

[56] Yadav RBS (2011). The 2007 Talala, Saurashtra, western India earthquake sequence. Tectonic implications and seismicity triggering. J Asian Earth Sci..

[57] Chiodini G (2004). Carbon dioxide Earth degassing and seismogenesis in central and southern Italy. Geophys. Res. Lett..

[58] Chiodini G (2010). Non- volcanic CO_2_ Earth degassing: Case of Mefite d’Ansanto (southern Apennines), Italy. Geoph. Res. Lett..

[59] Lucente FP (2010). Temporal variation of seismic velocity and anisotropy before the 2009 MW 6.3 L’Aquila earthquake, Italy. Geology.

[60] Savage MK (2010). The role of fluids in earthquake generation in the 2009 Mw 6.3 L’Aquila, Italy, earthquake and its foreshocks. Geology.

[61] Miller AS (2004). Aftershocks driven by high-pressure CO_2_ source at depth. Nature.

[62] Brune JN (1970). Tectonic stress and the spectra of seismic shear waves from earthquakes. J. Geophys. Res..

[63] Picozzi M (2019). Moment and energy magnitudes: diversity of views on earthquake shaking potential and earthquake statistics. Geophys. J. Int..

[64] Zaliapin I, Ben-Zion Y (2013). Earthquake clusters in southern California I: Identification and stability. J. Geophys. Res..

