# Supplementary material for: Detecting long-lasting transients of earthquake activity on a fault system by monitoring apparent stress, ground motion and clustering
Source: Sci Rep. 2019 Nov 7;9:16268. doi: 10.1038/s41598-019-52756-8 (PMC6838128; doi:10.1038/s41598-019-52756-8)
Supplement: Supplementary file 1 — Supplementary Information [file 41598_2019_52756_MOESM1_ESM.docx]

Detecting long-lasting transients of earthquake activity on a fault system by monitoring apparent stress, ground motion and clustering

Supplementary information

Picozzi M.^1^*, Bindi D.^2^, A. Zollo^1^, G. Festa^1^, and D. Spallarossa^3^

^1^University of Naples Federico II, Italy

^2^Helmholtz Centre Potsdam, GFZ German Research Centre for Geosciences, Germany

^3^University of Genova, Italy

*Corresponding author: matteo.picozzi@unina.it

**Introduction**

The Supplementary Information contains information about:

1. The estimation of radiated energy (E_S_) and seismic moment (M_0_) for microearthquakes
2. Scaled energy versus seismic moment – self-similarity
3. Scaling between seismic energy (E_S_) and seismic moment (M_0_)
4. Supplementary figures, 1 to 11.
5. Regression parameters of Eq. (2) and Eq. (3)
6. Regression parameters of Eq. (7)
7. References
8. **The estimation of radiated energy (E_S_) and seismic moment (M_0_) for microearthquakes**

We summarize here our procedure for the estimation of E_S_ and M_0_ for microearthquakes. We refer to Picozzi et al. (2019)^35^ for further details.

The procedure is based on calibrating empirical models which connect the source parameters to features extracted from the seismograms. The calibration data set is composed by 216 earthquakes in the magnitude range Mw 1.2 and Mw 3.2 occurred over the Irpinia fault. The source parameters for the calibration events were computed by Zollo et al. (2014)^9^ considering the following parametric model (reference):

$$M\left( f \right)=\frac{M_{0}}{1+\left( \frac{f}{f_{c}} \right)^{\gamma}}$$

where M_0_ is the seismic moment, fc is the corner frequency and γ is the high-frequency fall-off. Following Izutani and Kanamori (2001), we computed the radiated energy E_S_ for the calibration events by numerical integration of the theoretical source spectrum M(f), that is:

$$E_{S}=\frac{4\pi}{5\rho v_{S}^{5}}\int_{0}^{\infty} \left| fM\left( f \right) \right|^{2}df$$

The reliability of the source parameters has been discussed by Zollo et al. (2014)^9^. Regarding the seismic energy, considering a theoretical source model allows us to integrate over a wide range of frequency, avoiding the missing energy outside the frequency band as highlighted by Ide and Beroza (2001)^36^. Furthermore, the theoretical study on the reliability of estimates of source parameters of small earthquakes presented by Kwiatek and Ben-Zion (2016)^8^ indicates that, considering the sensor characteristics, the hypocentral distances, the magnitude range and the level of seismic noise at the ISNet network, reliable earthquake source parameters can be estimated for the range of magnitude analyzed by Zollo et al., (2014)^9^. Therefore, we consider reliable E_S_ and M_0_ estimated for the calibration earthquakes.

Following Picozzi et al. (2019)^35^, we consider two parameters measured on the S-wave signals in the time domain to calibrate empirical attenuation models to estimate the source parameters for the application data set. The two parameters are the integral velocity square (IV2_S_), used to estimate E_S_, and the peak in displacement (PD_S_), used to estimate M_0_. The empirical model for computing E_S_ from IV2_S_ is defined in equation (S1)

$log\left[ {IV2}_{S}\left( R_{H} \right) \right]=A+Blog\left( E_{S} \right)+w_{j}C_{j}+\left( 1-w_{j} \right)C_{j+1}$ (S1)

where the hypocentral distance R_H_ range is discretized into Nbin; the index j =1,..,Nbin indicates the j-th node selected such that R_H_ is between the distances rj≤ R_H_< rj+1; the attenuation function is linearized between nodes rj and rj+1 using the weights *w*, computed as *w*j = (rj+1 – R_H_)/(rj+1 – rj).

The R_H_ range 5-60 km is discretized into 12 bins with equal width (i.e., 5 km). The minimum distance is fixed to 5 km given the lack of recordings at shorter distances. The coefficients A, B, Cj are determined by solving the over-determined linear system (S1) in a least-square sense. To fix the trade-off between A and Cj, the attenuation is constrained to zero at r2=10 km.

Similarly, the relationship between the peak displacement PD and M_0_ has been expressed in the form:

$log\left[ {PD}_{S}\left( R_{H} \right) \right]=D+Flog\left( M_{0} \right)+w_{j}G_{j}+\left( 1-w_{j} \right)G_{j+1}$ (S2).

Figure S5 shows the attenuation models of Eqs. S1 and S2 (orange stars) in comparison with those obtained by Picozzi et al. (2019)^35^ in Central Italy (green stars).

The number of events in the calibration data set is only about 10% of the number of events in the application data set. However, we highlight that one should not look at the percentage, which obviously depends on the number of events in the application data set, but to the suitability of the calibration data set for constraining the empirical model in the magnitude range of interest (and for the region of interest). Figure S9 compares the distribution of the energy versus moment scaling of the calibration earthquakes (white squares with red contour) with the scaling relevant to the application data set (white stars with orange contour). In order to avoid extrapolation towards lower magnitudes, in the present study we only interpret results for seismic moment above 10^11^ Nm (blue stars). We consider the population in the calibration data set suitable to calibrate robust attenuation models over the energy and seismic moment application ranges of interest.

To show few examples of performance of the attenuation model for E_S_, in Figure S10 we present the single stations E_S_ estimates before and after the correction for the path. The plots a) and b) refer to events (stars with the same colour are different stations for the same event) with M_0_ between 11 Nm and 11.5 Nm, the lowest M_0_ available from Zollo et al. (2014)^9^. Plots c) and d) refer to events with M_0_ between 13 Nm and 13.5 Nm. We highlight that any kind of trend with distance is observed after the correction for the path.

1. **Scaled energy versus seismic moment – self-similarity**

Here we verify if the studied seismicity is self-similar.

As shown in Figure S2, the variation of the scaled energy (i.e., E_S_/M_0_) for two or three order of magnitude when represented as function of moment magnitude (i.e., non-self-similarity) is not uncommon. Most of the represented datasets shows a non-constant scaled energy with magnitude. We followed Kanamory and Rivera (2004, BSSA)^R1^ to investigate the source scaling by checking if the parameter ε in the scaling relation Mo ∝ fc-(3+ε) is different from zero.

We considered the E_S_ and M_0_ derived using IV2_S_ and PD_S_, the E_S_ and M_0_ estimates of the calibration dataset by Zollo et al. (2014)^9^, and those for Central Italy derived by Picozzi et al. (2019)^35^.

Figure S11 shows the normalized scaled energy with respect to the normalized seismic moment (blue stars, this study; red stars, Zollo et al., 2014^9^; orange stars Picozzi et al., 2019^35^) and the best-fit lines obtained parameterizing Eq. 15 of Kanamory and Rivera (2004, BSSA)^R1^. In all three cases, ε is different from zero (i.e., 0.3 this study; 0.31 Zollo et al., 2014^9^; 0.17 Picozzi et al., 2019^35^). The implications of our results is that large and small earthquakes occurring in the investigated areas have different rupture dynamics, as discussed by Kanamory and Rivera (2004, BSSA)^R1^.

1. **Scaling between seismic energy (E_S_) and seismic moment (M_0_)**

Figure S2b shows the existing scaling between seismic energy (E_S_) and seismic moment (M_0_), together with the E_S_-M_0_ best fit model (black line). Data have been coloured differently to emphasize that for a given M_0_ (i.e., for an average slip over the fault area), earthquakes can release different amount of seismic energy depending on the dynamic characteristics of the rupture process (e.g., stress drop, Δσ, and rupture velocity play a significant role). Similarly, earthquakes radiating the same amount of seismic energy can be associated to different deformation features (average slip and rupture dimension). The apparent stress τ_a_ (i.e., the ratio between E_S_ and M_0_ multiplied by the rigidity μ of the medium at the source location) contains the complementary nature of the information provided by E_S_ and M_0_ and is related to the stress-drop Δσ. Studying only M_0_ provides a partial view of the earthquake dynamic, being it a static measure of the rupture process. Differences in τ_a_ among earthquakes with equal M_0_ are very important to single out those associated to high rupture velocity and/or high Δσ (i.e., in the case of large earthquakes, τ_a_ can be exploited to characterize those with larger energy radiation and higher devastating power).

1. **Supplementary figures, 1 to 8.**

**Supplementary figure 1**


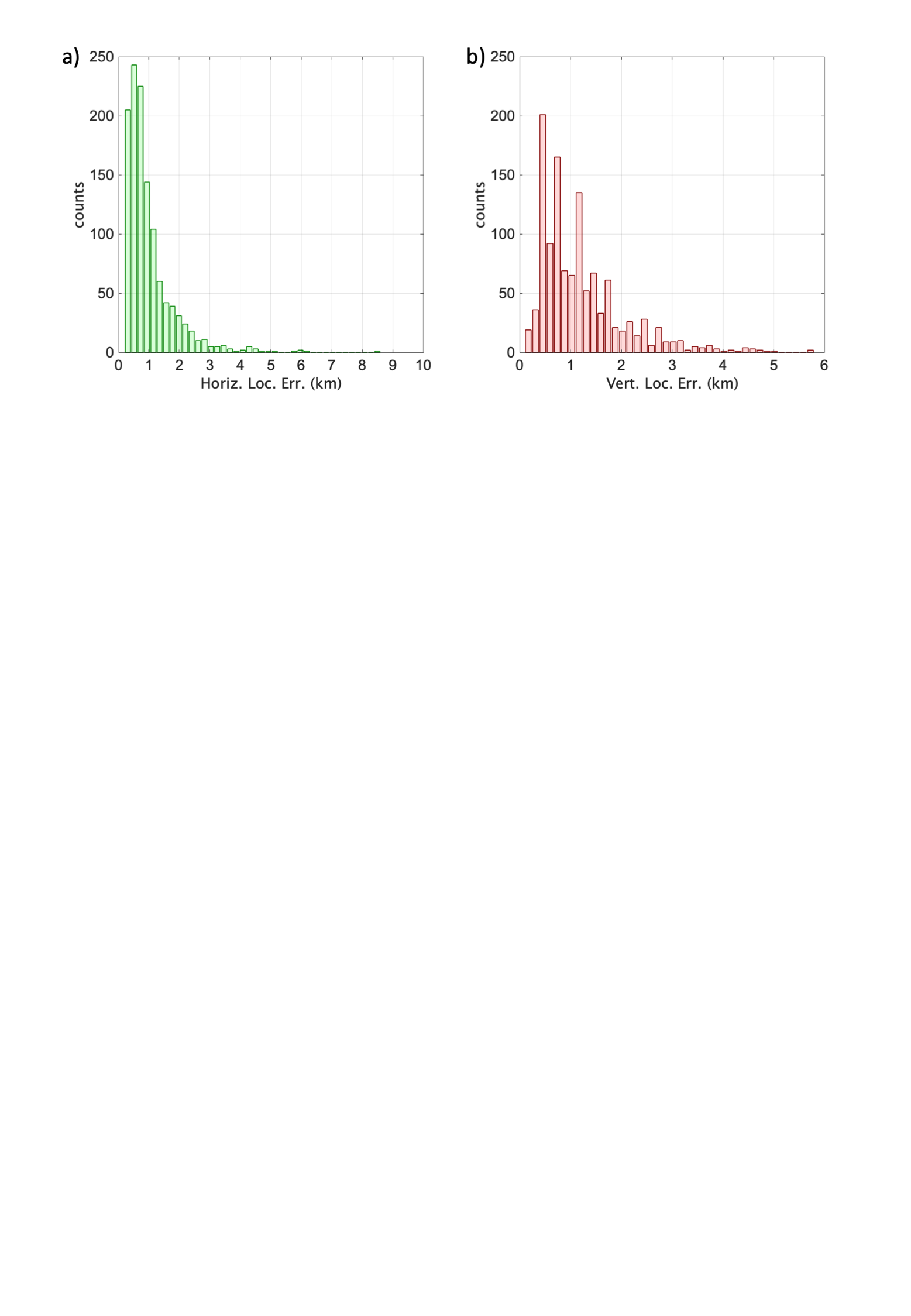


**Supplementary Figure S1:** (a) horizontal location errors. (b) vertical location errors.

**Supplementary figure 2**


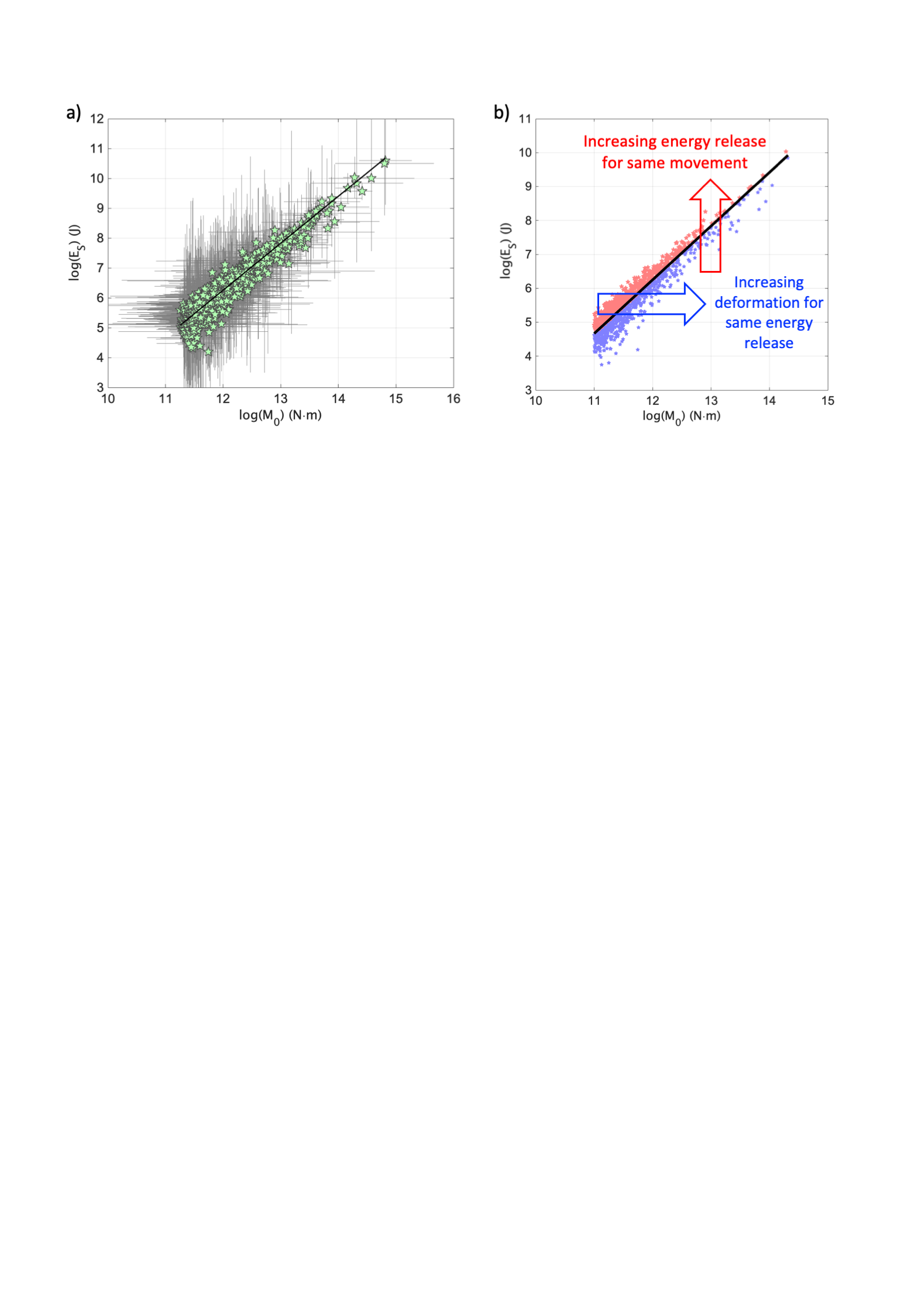


**Supplementary Figure S2:** (a) E_S_ versus M_0_ computed for the microearthquakes recorded at the ISNet seismic network (green stars, with ±1 standard deviations shown as vertical and horizontal bars); best-fit model (black line). (b) The same data of (a), but coloured according to their position with respect to the best-fit model: data above the model (red), below the model (blue).

**Supplementary figure 3**


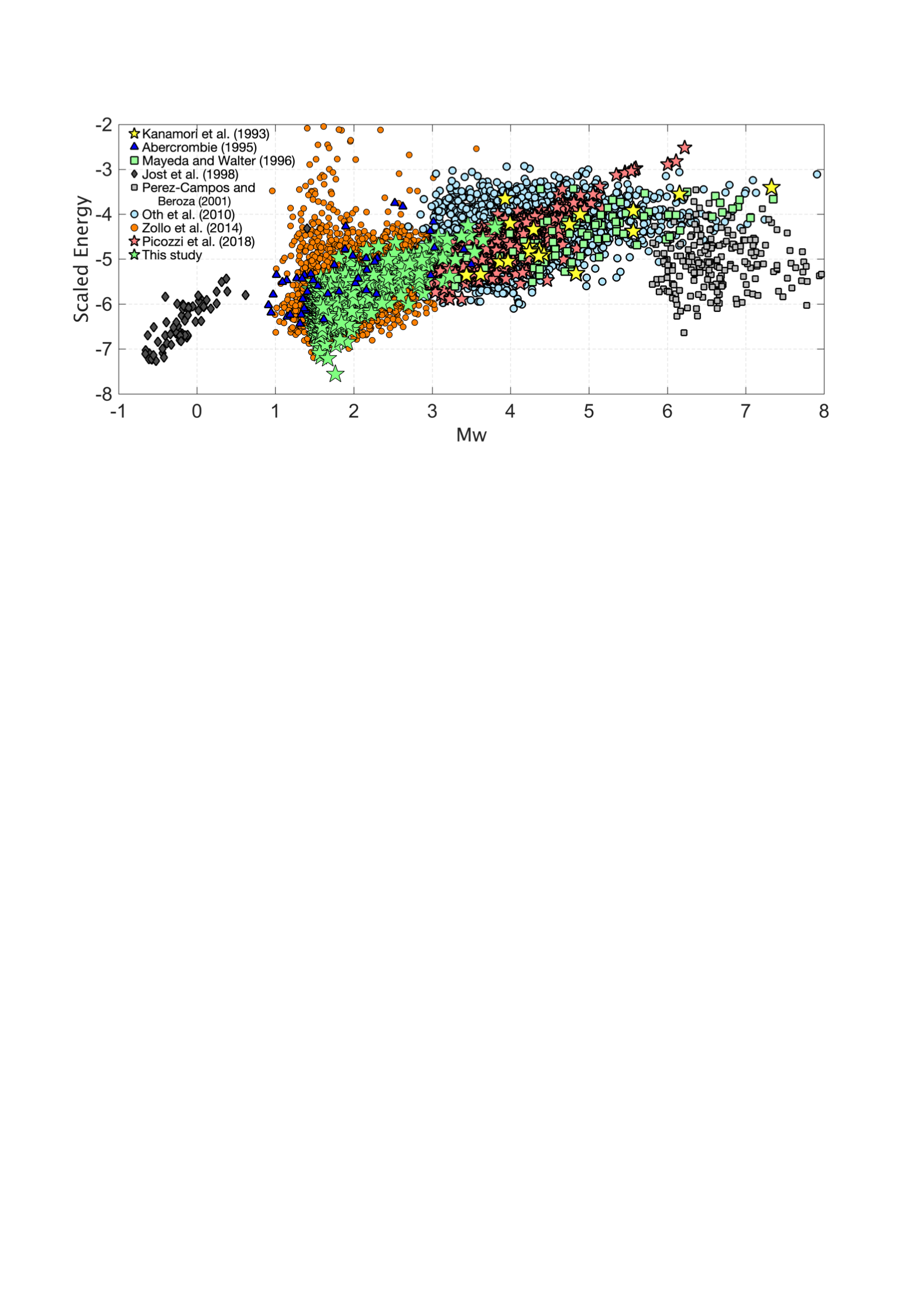


**Supplementary Figure S3:** Scaled energy versus moment magnitude for the dataset analysed in the present study (green stars) and eight additional other seismological data sets from tectonic areas worldwide involving a wide range of distances (i.e., from local to teleseismic) and seismic phases (e.g., direct P- and S-waves and coda)^R2-R5^.

**Supplementary figure 4**

**
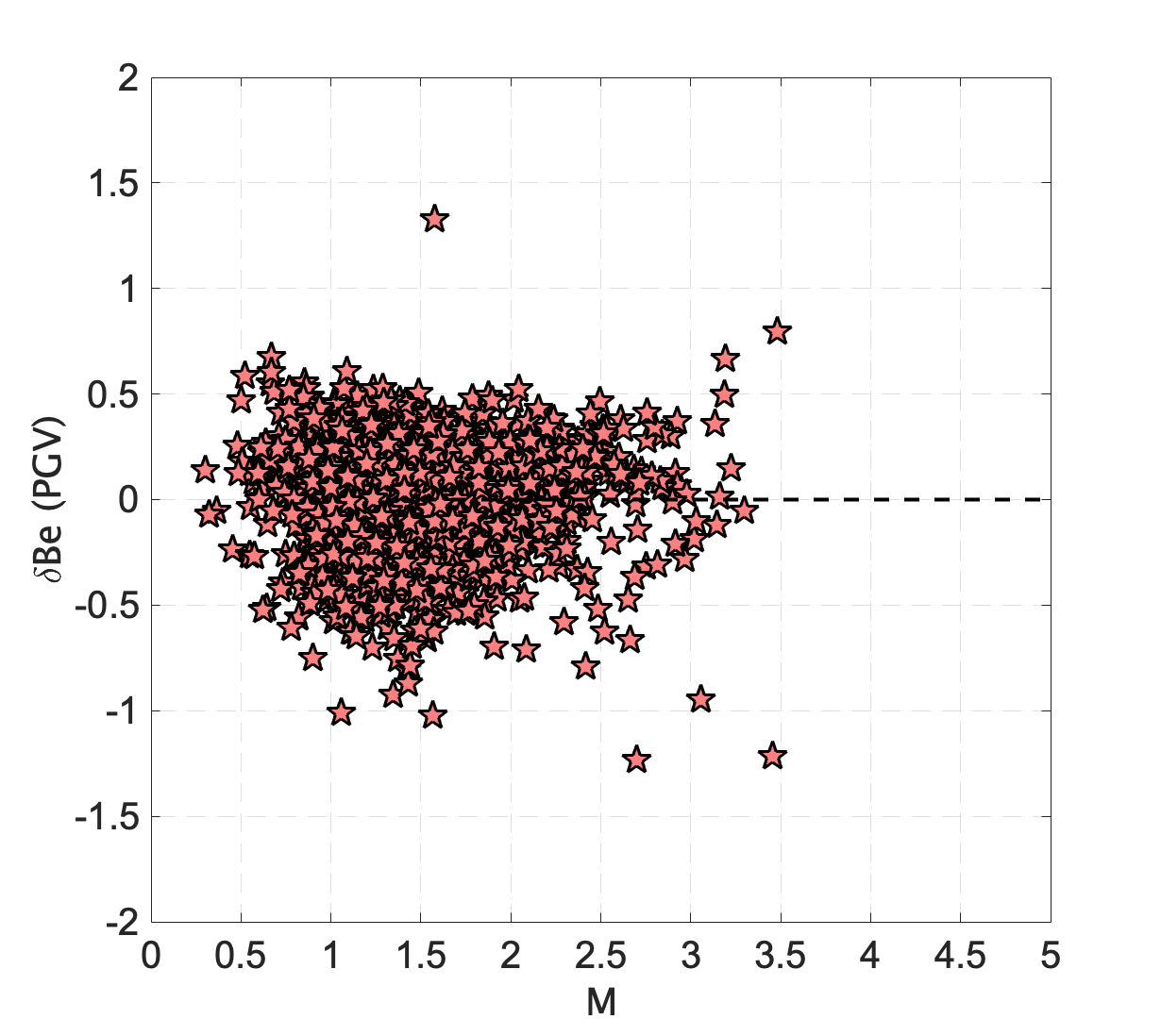
**

**Supplementary Figure S4:** δBe residuals for PGV versus magnitude; the zero-bias value (dashed line) is shown for reference.

**Supplementary figure 5**


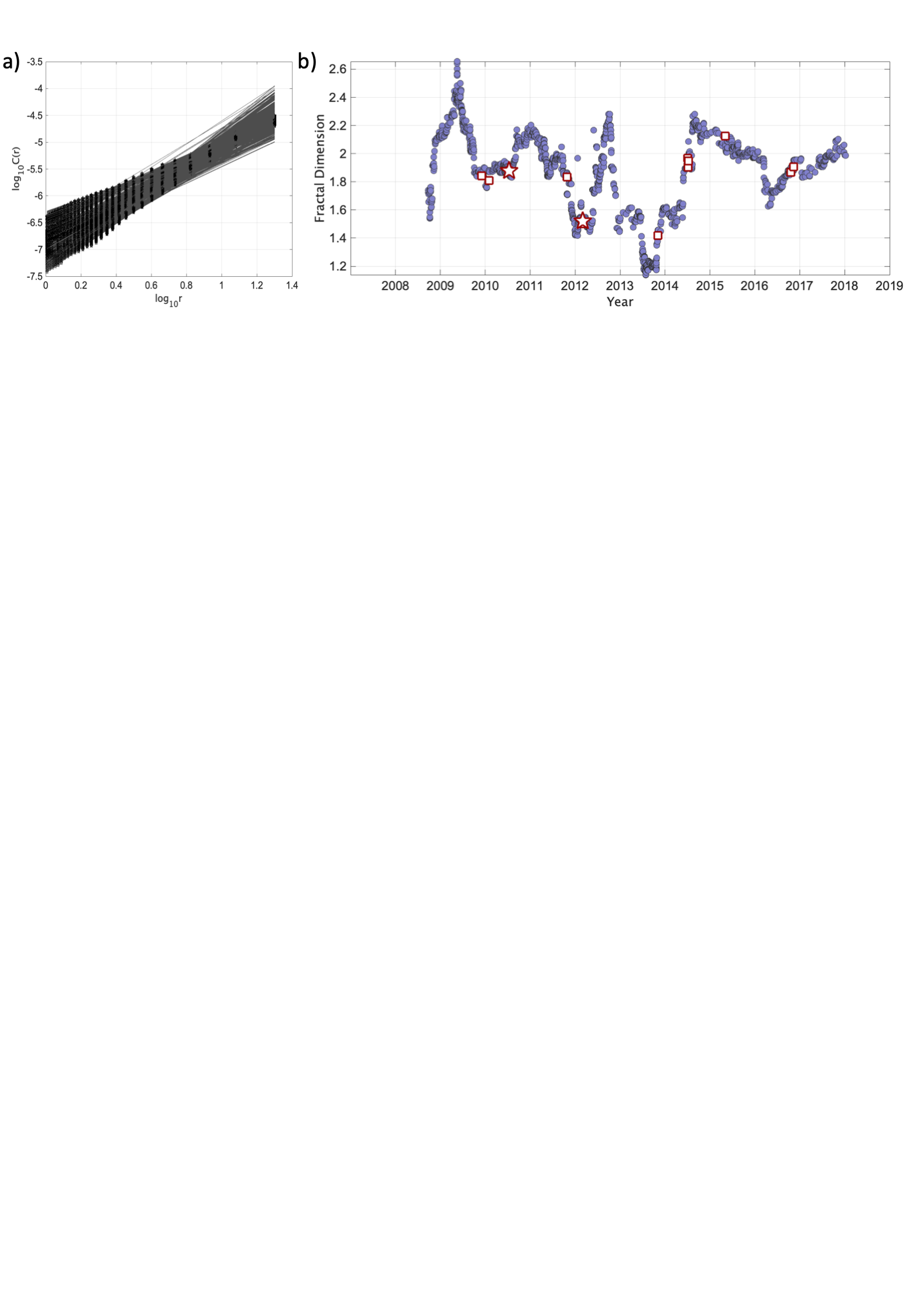


**Supplementary Figure S5:** (a) Plot of the correlations integral [log10(C(r)] against the radius of a sphere of investigation log_10_(r) for the 200-event windows (black points) together with best-fit models (grey lines). (b) Fractal dimension, *d*, versus time. Events with magnitude below Mw 3.0 (blue dots), with Mw >= 3.0 and Mw < 3.5 (white square with red contour), and those with Mw = 3.5 (white stars with red contour).

**Supplementary figure 6**

**Supplementary Figure S6:** (a) Plot of normalized Δτ_a_ (green triangles) and Δη (yellow dots) with and splines (lines). The vertical red dashed lines indicate the occurrence of the two Mw 3.5 earthquakes. (b) Splines as in (a), but after the trend removal. (c) histogram of the Spearman’s correlation.

**Supplementary figure 7**

**Supplementary Figure S7:** (a)The same as Figure_4_R2 but for normalized Δτ_a_ (green) and δBe (red) and splines.

**Supplementary figure 8**


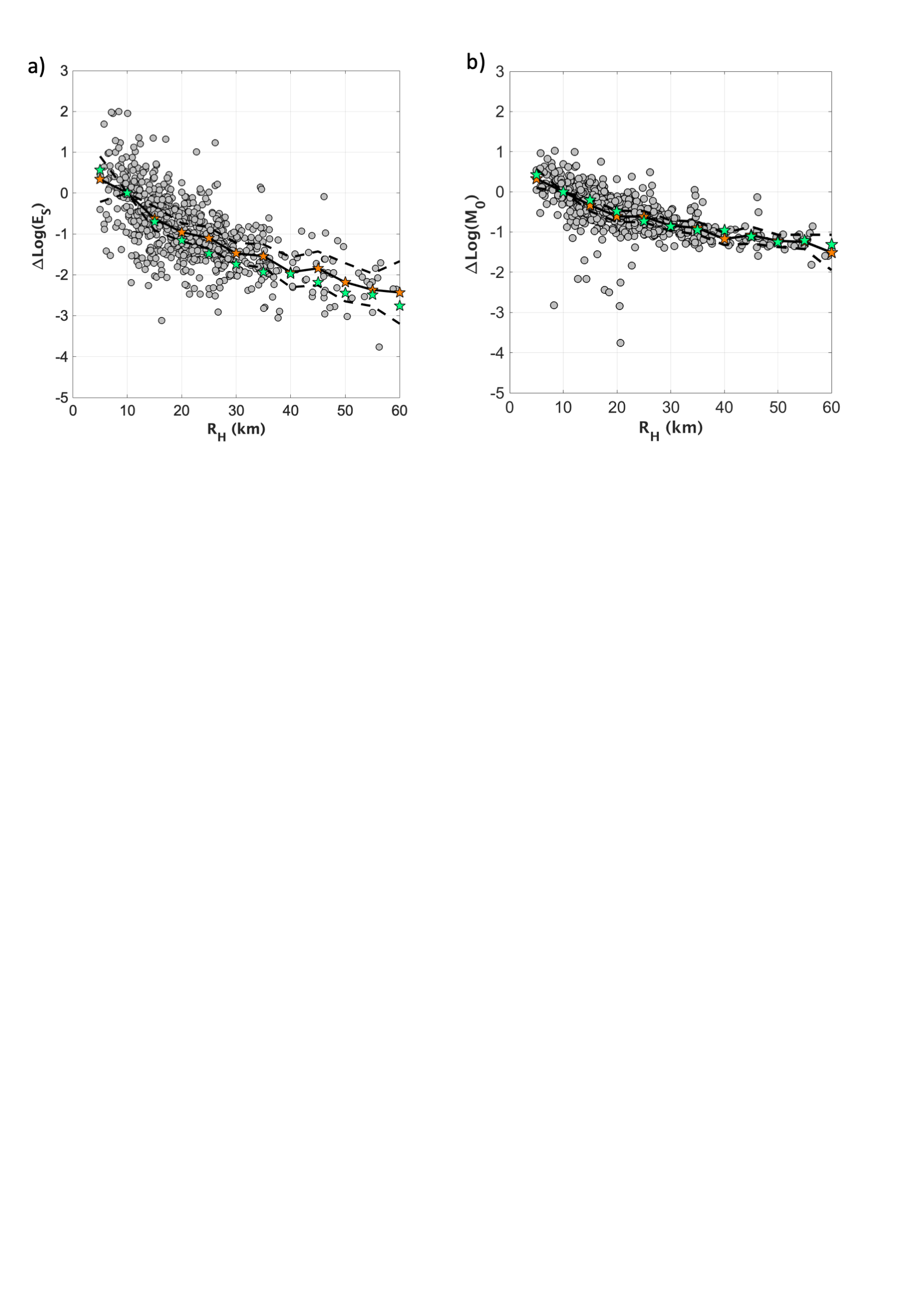


**Supplementary Figure S8:** (a) Results of the calibrations between IV2_S_ and E_S_ (Eq.2) and between PD_S_ and M_0_ (Eq.3). a) the coefficients C_j_ of Eq.2 (orange) are compared with the residuals $\Delta log\left( E_{S} \right)=log\left[ {IV2}_{S}\left( R_{H} \right) \right]-A-Blog\left( E_{S} \right)$ (grey circles); the coefficients C_j_ obtained for the Central Italy seismic sequence^35^ are also shown (green). (b) the coefficients G_j_ in Eq.3 (orange circles) are compared with the residuals $\Delta log\left( M_{0} \right)=log\left[ {PD}_{S}\left( R_{H} \right) \right]-D+Flog\left( M_{0} \right)$ (grey circles); the coefficients G_j_ obtained for the Central Italy seismic sequence^35^ are also shown (green). ±1 standard deviation associated to the C_i_ and G_j_ parameters (dashed black lines).

**Supplementary figure 9**


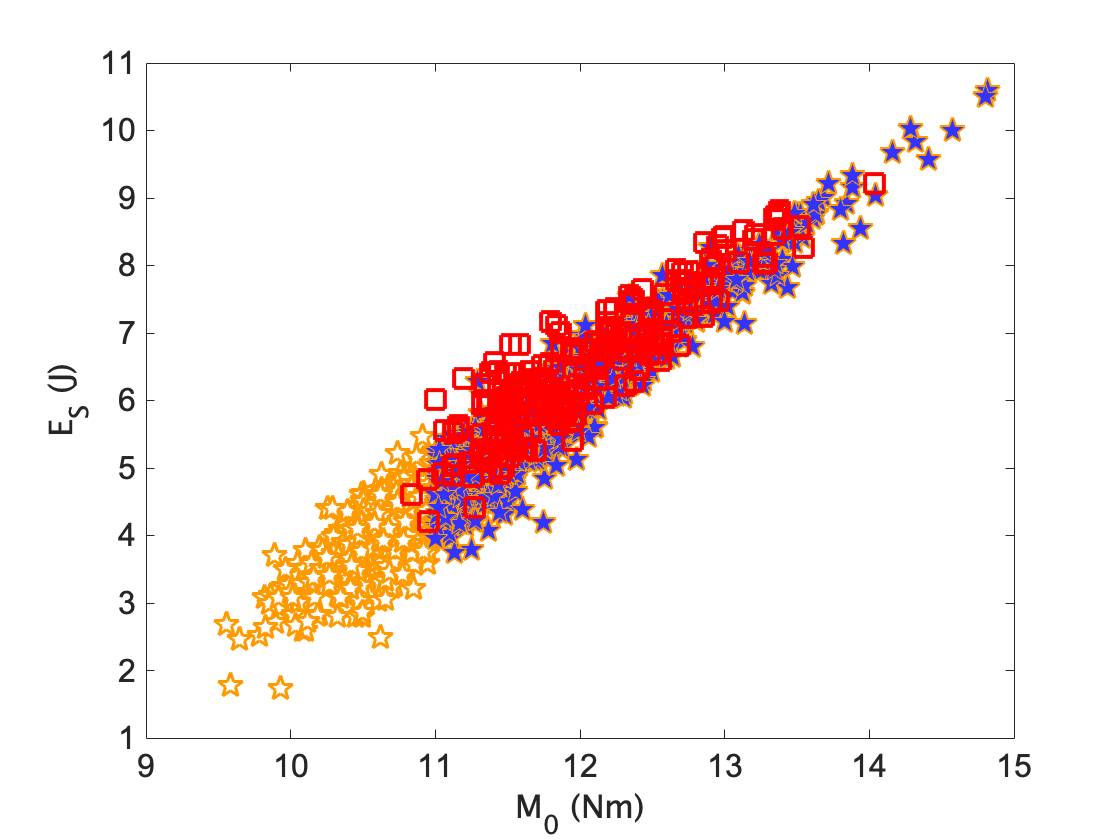


**Supplementary Figure S9:** E_S_ vs M_0_ for all the available events (orange stars), Zollo et al. (2014)^9^ (white squares with red contour), and those used to derive the apparent stress (blue stars).

**Supplementary figure 10**


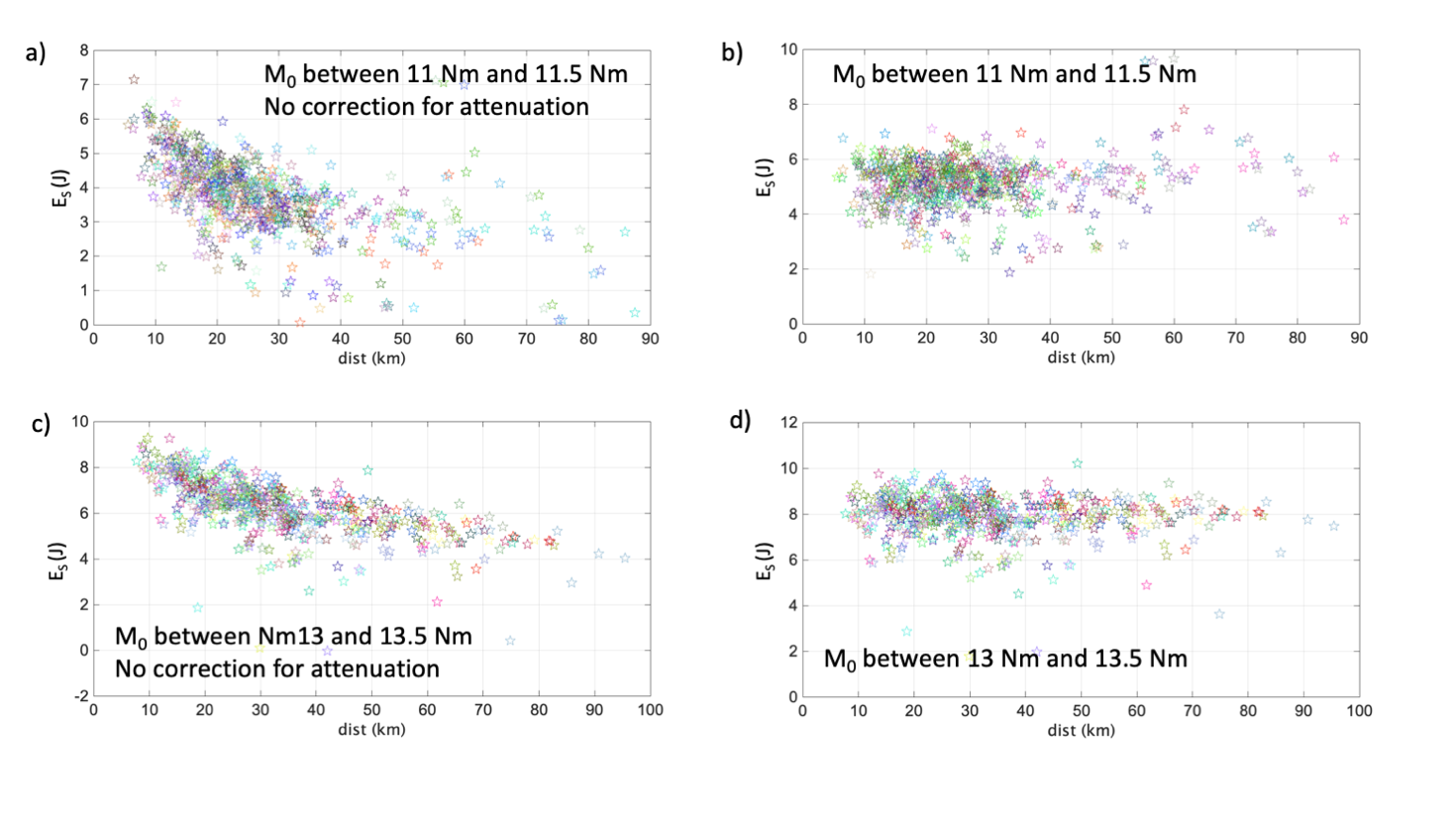


**Supplementary Figure S10:** Single stations E_S_ estimates before (a, c) and after (b, d) the path corrections. Stations with the same colour belong to the same event.

**Supplementary figure 11**


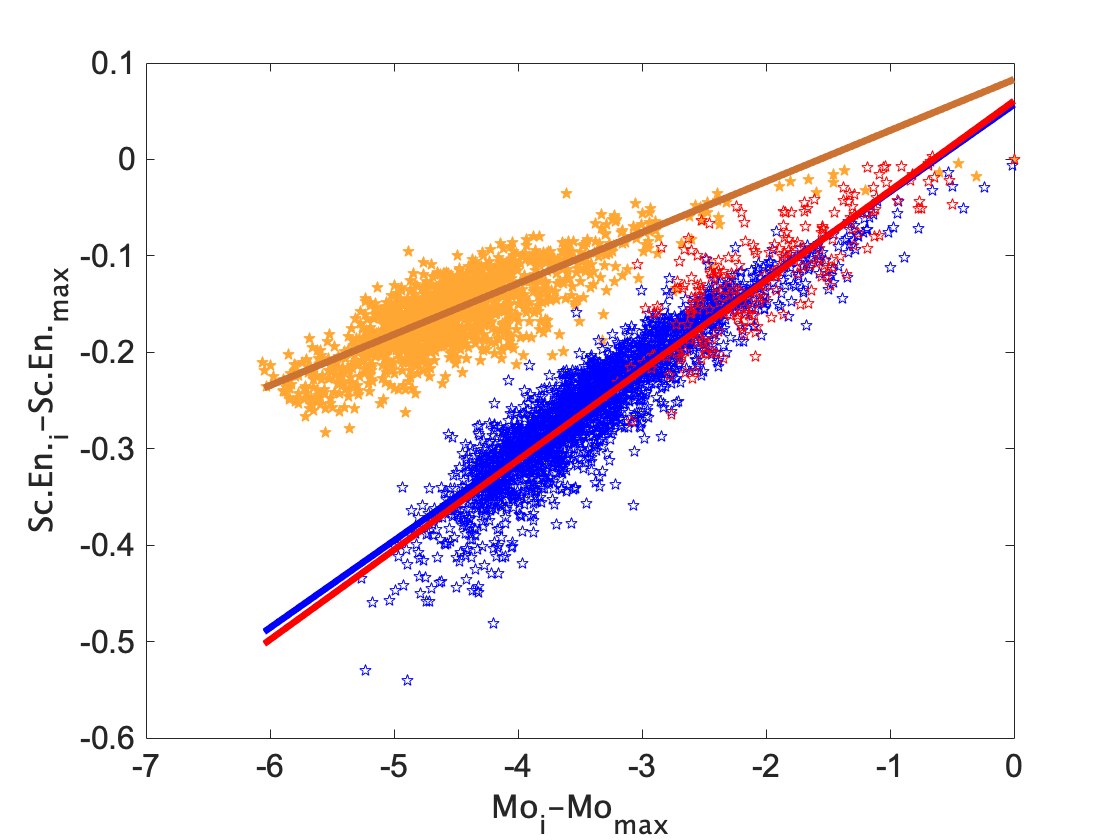


**Supplementary Figure S11:** Normalized scaling energy versus normalized seismic moment with best-fit lines for the dataset of this study (blue), Zollo et al.,2014^9^ (red), and Picozzi et al., 2019^35^ (orange).

1. **Regression parameters of Eq. (2) and Eq. (3)**

| **Parameters** | **Mean** ± **Stand. dev.** | | **Parameters** | **Mean** ± **Stand. dev.** | |
| --- | --- | --- | --- | --- | --- |
| **A** | -12.43 ± 0.18 | | **D** | -14.69 ± 0.42 | |
| **B** | 0.63 ± 0.03 | | **F** | 0.712 ± 0.035 | |
|  |  | |  |  | |
| **Parameters** | **Values** | **Stand. dev.** | **Parameters** | **Values** | **Stand. dev.** |
| **C_1_** | 0.343 | 0.285 | **G_1_** | 0.33 | 0.121 |
| **C_2_** | 0.0 | 0.0 | **G_2_** | 0.0 | 0.0 |
| **C_3_** | -0.618 | 0.148 | **G_3_** | -0.332 | 0.077 |
| **C_4_** | -0.957 | 0.114 | **G_4_** | -0.609 | 0.071 |
| **C_5_** | -1.101 | 0.135 | **G_5_** | -0.618 | 0.070 |
| **C_6_** | -1.476 | 0.132 | **G_6_** | -0.842 | 0.068 |
| **C_7_** | -1.541 | 0.145 | **G_7_** | -0.899 | 0.080 |
| **C_8_** | -1.936 | 0.186 | **G_8_** | -1.175 | 0.076 |
| **C_9_** | -1.841 | 0.211 | **G_9_** | -1.078 | 0.107 |
| **C_10_** | -2.180 | 0.238 | **G_10_** | -1.221 | 0.077 |
| **C_11_** | -2.367 | 0.203 | **G_11_** | -1.250 | 0.090 |
| **C_12_** | -2.431 | 0.390 | **G_12_** | -1.510 | 0.224 |

**Supplementary Table S1:** Regression parameters of Eq. (2) and Eq. (3) with their standard deviations for the calibration dataset.

1. **Regression parameters of Eq. (4)**

| A | B1 | B2 | C | Stand. dev. |
| --- | --- | --- | --- | --- |
| -4.36 | 1.11 | -0.11 | -1.31 | 0.23 |

**Supplementary Table S2:** Regression parameters of Eq. (4) and the standard deviation.

**Supplementary References:**

R1. Kanamori, H., and L. Rivera (2004), Static and dynamic scaling relations for earthquakes and their implications for rupture speed and stress drop, Bull. Seismol. Soc. Am., 94, 314–319.

R2. Oth, A., *et al.* Earthquake scaling characteristics and the scale-(in)dependence of seismic energy-to-moment ratio: Insights from KiK-net data in Japan. *Geophys. Res. Lett.* **37**, L19304, doi:10.1029/2010GL044572. (2010).

R3. Ide, S., & Beroza G. C. (2001), Does apparent stress vary with earthquake size? *Geophys. Res. Lett.* **28**, 3349-3352. (2001).

R4. Zollo, A., *et al.* Source parameter scaling and radiation efficiency of microearthquakes along the Irpinia fault zone in southern Apennines, Italy. *J. Geophys. Res. Solid Earth* **119**, 3256–3275, doi:10.1002/2013JB010116. (2014).

R5. Picozzi, M., Bindi, D., Spallarossa, D., Di Giacomo, D., Zollo A. (2019). A rapid response magnitude scale for timely assessment of the high frequency seismic radiation. Scientific Reports – Nature (2018) 8:8562 | DOI:10.1038/s41598-018-26938-9.
